# Electrochemical Bacterial Enrichment from Natural Seawater and Its Implications in Biocorrosion of Stainless-Steel Electrodes

**DOI:** 10.3390/ma13102327

**Published:** 2020-05-19

**Authors:** María José De La Fuente, Leslie K. Daille, Rodrigo De la Iglesia, Magdalena Walczak, Francisco Armijo, Gonzalo E. Pizarro, Ignacio T. Vargas

**Affiliations:** 1 Departamento de Ingeniería Hidráulica y Ambiental, Facultad de Ingeniería, Pontificia Universidad Católica de Chile, Vicuña Mackenna 4860, Macul, Santiago 7820436, Chile; mjdelafu@uc.cl (M.J.D.L.F.); gpizarro@ing.puc.cl (G.E.P.); 2 Departamento de Genética Molecular y Microbiología, Facultad de Ciencias Biológicas, Pontificia Universidad Católica de Chile, Portugal 49, Santiago 8330025, Chile; lkdaille@uc.cl (L.K.D.); rdelaiglesia@bio.puc.cl (R.D.l.I.); 3 Departamento de Ingeniería Mecánica y Metalurgia, Facultad de Ingeniería, Pontificia Universidad Católica de Chile, Vicuña Mackenna 4860, Macul, Santiago 7820436, Chile; mwalczak@ing.puc.cl; 4 Departamento de Química Inorgánica, Facultad de Química y de Farmacia, Pontificia Universidad Católica de Chile, Vicuña Mackenna 4860, Macul, Santiago 7820436, Chile; jarmijom@uc.cl; 5 Marine Energy Research & Innovation Center (MERIC), Avda. Apoquindo 2827, Santiago 7550268, Chile

**Keywords:** microbial enrichment, overpotential, marine biocorrosion, stainless steel

## Abstract

Microbial electrochemical technologies have revealed the opportunity of electrochemical enrichment for specific bacterial groups that are able to catalyze reactions of interest. However, there are unsolved challenges towards their application under aggressive environmental conditions, such as in the sea. This study demonstrates the impact of surface electrochemical potential on community composition and its corrosivity. Electrochemical bacterial enrichment was successfully carried out in natural seawater without nutrient amendments. Experiments were carried out for ten days of exposure in a closed-flow system over 316L stainless steel electrodes under three different poised potentials (−150 mV, +100 mV, and +310 mV vs. Ag/AgCl). Weight loss and atomic force microscopy showed a significant difference in corrosion when +310 mV (vs. Ag/AgCl) was applied in comparison to that produced under the other tested potentials (and an unpoised control). Bacterial community analysis conducted using 16S rRNA gene profiles showed that poised potentials are more positive as +310 mV (vs. Ag/AgCl) resulted in strong enrichment for Rhodobacteraceae and *Sulfitobacter*. Hence, even though significant enrichment of the known electrochemically active bacteria from the Rhodobacteraceae family was accomplished, the resultant bacterial community could accelerate pitting corrosion in 316 L stainless steel, thereby compromising the durability of the electrodes and the microbial electrochemical technologies.

## 1. Introduction

Microbial electrochemical technologies (METs) take advantage of microbial communities to catalyze redox reactions over electrodes [1,2,3,4]. Even though the first generation of METs were designed to produce electricity, their efficiency as wastewater treatment systems has also been proven [3,5,6,7]. The next generation of METs were then conceived to accomplish selective and energy-efficient treatment, remediation, and recovery of nutrients, metals, and by-products with industrial value [8]. However, many of these novel and disruptive METs have only been tested at the laboratory scale and under controlled conditions. Therefore, some of the main ongoing challenges for METs are to overcome the practical limitations associated with (i) the performance requirements of cost-efficient materials used as electrodes, and (ii) the enrichment of specific microbial communities which can catalyze reactions of interest in a MET under natural, uncontrolled environmental conditions [9,10], which is related to the use of METs in the implementation of bioremediation strategies.

The durability of electrode materials under exposure to aggressive natural environments where complex microbial communities can develop (e.g., coastal areas) is a major concern for the scaling-up of METs. Due to their biocompatibility and corrosion resistance, carbon-based materials (e.g., graphite and carbon felt) are commonly used as electrode materials in laboratory scale METs. However, in pilot systems, they exhibit limitations in terms of resistivity, mechanical strength, and cost [10,11,12]. Stainless steel (SS) is a common industrial material characterized by its good mechanical properties, corrosion resistance, conductivity, cost efficiency, and easy scalability [13,14,15]. Pocaznoi et al. (2012) [16] compared the abilities of carbon cloth, graphite plate, and SS as bioanodes in bioelectrochemical reactors (BERs), concluding that SS electrodes presented the highest current density among the tested materials. Furthermore, SS electrodes have been successfully proven to be effective as both anodes and cathodes in marine METs [14], producing power levels similar to those produced with graphite electrodes (0.2 W m^−2^) [17]. Thus, although the material evaluation of METs under real service conditions is in its infancy, SS emerges as a good candidate as an electrode material for the scaling-up of METs in marine environments.

Poised potential experiments have been used to study the effect of electrode potential on microbial colonization of the electrode in vitro [9,18,19,20,21]. This strategy has been proven by several authors to be able to modulate and enrich the presence of particular electrochemically active bacteria (EAB) which can transform contaminants in a BER. Indeed, while negative potentials have been shown to enrich heterotrophic exoelectrogenic bacteria [18,21], the use of a positive potential tends to enrich autotrophic microorganisms that are able to directly or indirectly receive electrons from an electrode [20]. In general, these types of experiments have been developed in culture media under controlled conditions using an inoculum obtained from wastewater treatment plants or anaerobic soil, where the microorganisms of interest are found in high abundance [22]. In consequence, it is necessary to evaluate the effectiveness of the application of an overpotential to an electrode as a tool to enrich the presence of EAB in METs. To determine if this tool could help with the scalability and applicability of MET in real environments, this evaluation should be accomplished under exposure to complex natural environments such as coastal marine environments.

In this work, we investigated whether the durability of SS electrodes exposed to a marine environment changes according to the applied overpotential and the enrichment of specific bacteria, as well as the application of an overpotential as a microbial enrichment tool to improve the applicability and scalability of MET. To address the proposed objective, a complete and multi-technique approach was used to evaluate the effect of different overpotentials on both the corrosion and the biological colonization of SS as an electrode material for METs operating in a coastal marine environment. Our results demonstrated differential electrochemical bacterial enrichment under the different overpotentials tested. In particular, the highest positive potential led to clear enrichment in members of the *Roseobacter/Rhodobacter* clade, and exerted a strong effect on deterioration of the SS electrodes.

## 2. Materials and Methods

### 2.1. Reactor Configuration

In this work, two sets of experiments were conducted. The first experiment was carried out to determine the effect of overpotential application on microbial enrichment from natural seawater samples, and the second experiment was conducted to isolate the effects of both the applied potential (abiotic) and the enriched microorganisms (biotic) on the metal corrosion.

**Figure 1:**
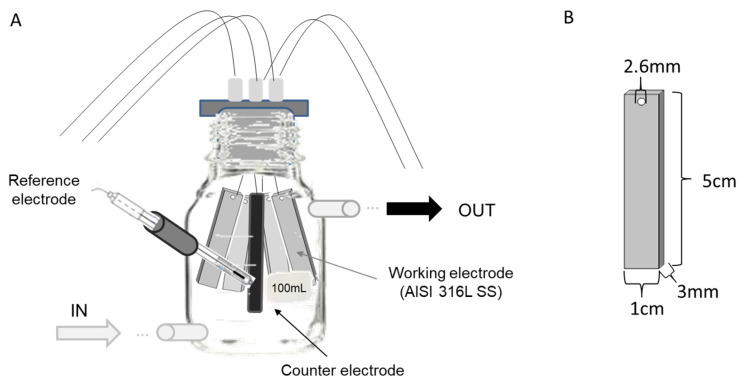
Experimental design. (**A**) Design of the bottles, indicating seawater inlet and outlet and the arrangement of the AISI 316L Stainless Steel (SS) electrodes, reference electrode, and counter electrode. (**B**) Dimensions of SS electrodes.

The first experiment was run using four 100 mL glass bottles as three-electrode electrochemical cells, which were modified to allow a continuous closed flow of natural seawater during the experiment (Figure 1). Each bottle contained four AISI 316L (X2CrNiMo17-12-2, material number 1.4404) SS electrodes as the working electrode (1.5 cm^3^ each), one graphite rod as the counter electrode (2 cm^3^), and one Ag/AgCl electrode as the reference electrode (3 M KCl) (Figure 1). This experimental design was based on the reactor designed by [21]. Before seawater exposure, each SS plate was polished using emery paper (grit 240), rinsed with distilled water, degreased with acetone, and finally dried by blowing hot air. An Interface 1000TM potentiostat (GAMRY, Warminster, PA, USA) connected to an ECM8™ electrochemical multiplexer (GAMRY, Warminster, PA, USA) was used to poise each cell at one of three specific potentials over the ten-day course of the experiments. The potentials were: (i) −150 mV (vs. Ag/AgCl), representing the reported optimal applied potential for enriching marine EAB [9]; (ii) +100 mV (vs. Ag/AgCl), corresponding to the open circuit potential (OCP) reported for 316L SS under marine conditions after 10 days [23] and allowing for the enrichment of autotrophic exoelectrotrophic microorganisms on the electrodes [20]; and (iii) +310 mV (vs. Ag/AgCl), representing a near potential of one of most-positive potentials reported for the oxygen reduction reaction [24]. As a control, a fourth electrochemical cell was run under OCP conditions.

The second experiment involved the same experimental setup as that used in the first, but two cells were set under abiotic conditions, with 0.2 µm-filtered natural seawater. Inside of one of these two cells the working electrode was polarized at +310 mV (vs. Ag/AgCl) and the other was run as the control under OCP conditions. A third electrochemical cell was tested under biotic conditions (using unfiltered seawater) and the working electrode was poised at +310 mV (vs. Ag/AgCl).

For both experiments, fresh, natural coastal seawater was collected from the Estación Costera de Investigaciones Marinas (ECIM) of the Pontificia Universidad Católica de Chile, located in the Eastern Pacific Ocean at 33°30′16′′ S, 71°38′23′′ W. Water was stored in a temperature-controlled room at 20 °C. Air pumps and a light schedule (12 h/12 h) were used to maintain saturated conditions of phototrophic activity and dissolved oxygen (8 mg L^−1^) during the experiment. Electrochemical cells were connected to a tank in a closed-loop system. A peristaltic pump was used to maintain a constant flow of seawater (0.5 mL s^−1^) throughout the 10 days of the experiment.

### 2.2. Surface Analysis for Corrosion Evaluation

#### 2.2.1. Weight Loss

To assess weight loss, each coupon was weighed before the assembly of the experiments with an analytical balance with a precision of 0.1 mg (Shimadzu ATY224, Kyoto, Japan). The samples analyzed were the three SS electrodes after they were first sonicated for community analysis (described in Section 2.3). After sonication, the SS electrodes were washed, degreased with acetone, and dried with hot air to be reweighed. A one-way analysis of variance (ANOVA), followed by an a posteriori Tukey test, were performed using GraphPad Prism version 6 to check for significant differences between treatments [25].

#### 2.2.2. Atomic Force Microscopy (AFM)

For the second experiment, after the cleaning of coupons to remove the biofilm, the topographies of the surfaces of the SS electrodes were observed using atomic force microscopy (AFM). The measurements were performed with an Innova^®^ Atomic Force Microscope (Bruker, Billerica, MA, USA). Each coupon was measured in triplicate at different points to obtain a representative analysis. The topography was measured in air, using the tapping mode with a silicon nitride probe. Roughness analysis was carried out on the images obtained, over an area of 35 mm × 35 mm.

### 2.3. Microbial Community Analysis

#### 2.3.1. DNA Extraction

After the exposure time, three of the four SS electrodes in each bottle were removed and sonicated in 50 mL of sterile seawater for 5 min (Elmasonic S 30H, Elma Schmidbauer GmbH, Frechen, Germany). The sonicated products were filtered through a 0.2-µm membrane filter to collect the biomass and perform the subsequent DNA extraction with phenol:chloroform based on a previously reported protocol [26]. Additionally, at the end of the experiment, 2 L of water from the tank was filtered through a 0.2-µm membrane filter to assess changes in the microbial community of the water column present in the tank. DNA quantification was performed with a Qubit^®^ 2.0 fluorometer (Invitrogen^TM^, Life Technologies, Carlsbad, CA, USA) according to the manufacturer’s protocol.

#### 2.3.2. Fragment Analysis (FA) of 16S rRNA Genes

To identify the microbial community composition developed on the tested electrodes, amplification of 16S rRNA genes was performed in all DNA samples. The 16S rRNA genes were amplified by polymerase chain reaction (PCR), using the 5′-NED-labeled primer 27F (5′-AGAGTTTGATCMTGGCTCAG-3′) and the primer 1492R (5′-GGYTACCTTGTTACGACTT-3′), without fluorescent labels [27]. The final concentrations in the PCR mix were 1.2 mM MgCl_2_, 1X KAPA PCR buffer B (KB1002), 0.3 mM dNTPs, 0.3 μM for each primer, and 1 U KAPA Taq polymerase (KAPABiosystems, Cape Town, South Africa). PCR conditions were as follows: 95 °C for 5 min, 25 cycles of 95 °C for 45 s, 56 °C for 45 s, and 72 °C for 1 min, and a final elongation step at 72 °C for 7 min. 16S rRNA gene PCR amplicons (1492 bp in length) were checked by electrophoresis on a 1% agarose gel. PCR products (17.5 μL) were digested independently with 2.5 U of *Hae*III restriction endonuclease and incubated overnight at 37 °C. Terminal restriction fragments (T-RFs) were detected using capillary electrophoresis (Macrogen, Seúl, Korea), and data were obtained using Peak Scanner software (v1.0, AB Applied Biosystems, Foster City, CA, USA). The sizes of the T-RFs were determined using the internal standard LIZ1200 (Applied Biosystems, Rotkreuz, Switzerland). Data analysis included the T-RFs between 50 and 900 nucleotides and excluded those representing less than 0.5% of the total area/abundance [28], thus recalculating the area of each selected T-RF as a proportion of the total area.

#### 2.3.3. 16S rRNA Gene Clone Library Construction

To identify the different operational taxonomic units (OTUs) observed in the Terminal Restriction Fragment Length Polymorphism (T-RFLP) profiles, a clone library was created for the 16S rRNA gene. For this purpose, amplification and subsequent cloning of the 16S rRNA gene were performed from the DNA of each sample using primers 27F (5′-AGAGTTTGATCMTGGCTCAG-3′) and 1492R (5′-GGYTACCTTGTTACGACTT-3′) [27]. The PCR products obtained were cloned into the vector TOPO-TA using a mixture containing 4 µL of PCR product, 1 µL of salt solution, and 1 µL of cloning vector (Cloning Kit R for Sequencing, Invitrogen). This mixture was incubated at room temperature for 20 min, and then 45 µL of competent cells of *Escherichia coli* (DH5α) were added and incubated in ice for 20 min. The mixture was then placed in the thermoregulated bath at 42 °C for 50 s and finally placed in ice for 2 min. After transformation, the mixture was incubated in 950 µL of Super Optimal Broth (SOB) medium (20 mM glucose, 20 g L^−1^ tryptone, 5 g L^−1^ yeast extract, 0.5 g L^−1^ NaCl, 2.5 mM KCl, 5 mM MgCl_2_, and 5 mM MgSO_4_, pH 7.0) for 1.5 h at 37 °C with constant agitation. Afterward, 100 µL of the incubated solution was collected and seeded on LB agar plates supplemented with ampicillin (50 µg mL^−1^), 10 µL of X-gal (80 mg mL^−1^), and 40 µL of IPTG (500 mM). The seeded plates were incubated at 37 °C overnight.

To corroborate the presence of the fragment of interest in the cloning vector, cloned sequences were amplified by PCR using the M13 forward (M13F) (5′-GTAAAACGACGGCCAG-3′) and M13 reverse (M13R) (5′-CAGGAAACAGCTATGAC-3′) primers. The final concentrations in the PCR mix (final volume 25 µL) were: 1.2 mM MgCl_2_; 1X buffer without Mg^2+^; 0.25 mM dNTPs; 0.5 μM each of M13F and M13R; and 1.25 U KAPA Taq polymerase. PCR conditions were: 94 °C for 10 min; 25 cycles at 94 °C for 45 s; 50 °C for 1 min; 72 °C for 1 min; and a final elongation step at 72 °C for 7 min. PCR amplicons were checked by electrophoresis on a 1.5% agarose gel. Positive clones were confirmed based on their length of about 780 bp. Positive clones were sequenced at Macrogen Inc., Seoul, Korea. The obtained clone sequences are available in the Sequence Read Archive database (Table S1).

#### 2.3.4. Analysis of Clone Sequences

Sequence Scanner Software v1.0 (AB Applied Biosystems, Foster City, CA, USA) was used to check the quality of the sequences. Sequences were then analyzed with VECTOR NTI software Version 11.5.4 (Thermo Fisher Scientific, Waltham, MA, USA) to eliminate the remaining vector sequence and leave the sequences in the 5′–3′ direction, considering the primer 27F as the beginning. Through this software, an in-silico analysis was also performed with the restriction enzyme *Hae*III for the subsequent taxonomic assignment of the observed T-RFs. The T-RFs of each condition were assigned with the in-silico analysis of the sequenced clones for each condition, except in the +100 mV (vs. Ag/AgCl) condition. In this condition no clones were obtained, and only taxonomic assignment was made using the T-RFs. All edited sequences were compared to the published database (nr/nt) using BLASTn [29] from the National Center for Biotechnology and Information (NCBI, December 2018).

#### 2.3.5. Amplicon Analysis of 16 rRNA Gene Sequences

To obtain a deeper taxonomic analysis of the microbial community enriched for under each set of conditions, amplicon analysis (AA) using next-generation sequencing (NGS) of the V4 hypervariable region of the 16S rRNA gene was performed with the Illumina MiSeq platform (Integrated Microbiome Resource (IMR), Toronto, ON, Canada). The sequences obtained in the present study are publicly available in the Sequence Read Archive database under the accession number PRJNA603906. The 16S rRNA gene amplicon sequences were processed using Mothur [30]. Sequences were de-multiplexed, assembled, and assigned to samples by matching them to barcode sequences using the *make.contigs* script and primers were removed using *cutadapt* [31]. Sequences with undesired lengths (200–300 bp), ambiguous nucleotides, and homopolymers longer than 8 bp were removed before further analysis. Afterward, sequences were aligned using the recreated Silva SEED v119 [32] as the reference. Chloroplast, eukaryotic, archaeal, and mitochondrial sequences were discarded. Sequences were also checked for PCR chimeras using UCHIME version 4.2.40 (mybiosoftware) [33]. High-quality sequences were clustered into operational taxonomic units (OTUs) with the furthest-neighbor algorithm, with a minimum sequence identity cut-off of 97%. Taxonomic assignments were performed against Silva v119 [32]. OTUs formed by 20 or fewer reads were not considered in subsequent analyses. For the OTUs that could not be identified at the gender level, a manual BLASTn was performed, wherein each sequence was compared to the published database (December 2018) (nr/nt) using BLASTn [29] from NCBI.

#### 2.3.6. Statistical Analysis of Fragment and Amplicon Analysis Profiles

To determine the similarity between the samples and the robustness of the results obtained by FA and AA, fragment and amplicon profiles were statistically analyzed via multivariate analysis using Primer-E software version 6 (Primer-E, Plymouth, UK). For each dataset (FA and AA), the average of the relative abundance profile of each replicate of the OTU was transformed to its square root, and then a similarity matrix was obtained using the Bray–Curtis coefficient [34]. With the similarity matrix thus generated, a cluster was performed for visual interpretation of the grouping, where the proximity between samples corresponded to their similarity. To evaluate the statistical significance of the differences between and within communities, a one-way ANOSIM and a SIMPROF analysis were performed [35].

#### 2.3.7. Similarity Analysis between Cloning and Amplicon Sequences

The sequences obtained via cloning and amplicon sequences were compared to determine the similarity between sequences and therefore the robustness of the community composition results. For this, the sequences of the most abundant OTUs, associated with the same bacterial genera in cloning and amplicon sequences, were used to perform a BLAST between sequences. With these results, a literature search was carried out to identify the metabolism and natural environment reported for each detected genus.

To determine the difference and/or similarity in bacterial identification and metabolic inference between samples, main bacterial taxa were identified using 16S rRNA gene sequences of clone library and AA results. Each sequence was taxonomically assigned by manual BLASTn of the sequence against the NCBI nt/nr database. For this analysis, only the families with a relative abundance higher than 1% of the total abundance in each sample were used.

#### 2.3.8. Microscopy Analysis

The SS coupons used for microscopy were cut into two parts. One part was used for epifluorescence microscopy and the other for scanning electron microscopy (SEM). For epifluorescence microscopy, each coupon was immersed in 0.1% w/v acridine orange (Sigma Chemical Co., St. Louis, MO, USA) for 5 min at room temperature. Then, each coupon was washed with Phosphate Buffered Saline (PBS). Each coupon was examined using an Olympus CX31 microscope (Olympus, Tokyo, Japan). The other halves of the coupons were used for SEM analysis. For SEM analysis, samples were fixed with 2% glutaraldehyde, treated by critical-point drying, and coated with a thin gold film. Each coupon was examined using a LEO 1420VP microscope (LEO Electron Microscopy Inc., New York, NY, USA) [23]. For each coupon, three pictures were taken at randomly selected areas.

## 3. Results and Discussion

### 3.1. Electrode Deterioration

**Figure 2:**
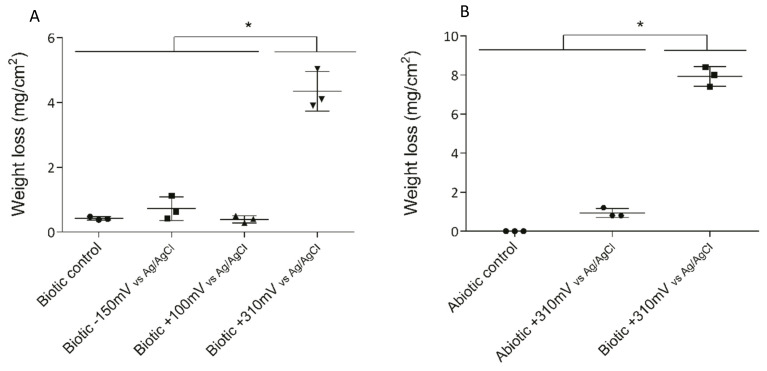
Weight loss of SS electrodes under each treatment after 10 days. To determine the significant differences between the treatments, a posteriori Tukey test was performed. Significant differences between treatments are indicated with asterisks, where each asterisk group treatments according to similarity. (**A**) Weight losses in the initial experiment under biotic conditions. (**B**) Weight losses under the abiotic and biotic conditions with the +310 mV (vs. Ag/AgCl) potential applied.

Three different potentials were applied to the 316L SS electrodes to determine the effect of overpotential application on electrode deterioration and bacterial enrichment. After 10 days of exposure, the maximum weight loss (as an indicator of deterioration) was observed for the +310 mV (vs. Ag/AgCl) treatment, with significant differences (*P* < 0.05) from that which occurred under the other conditions (Figure 2A).

**Figure 3:**
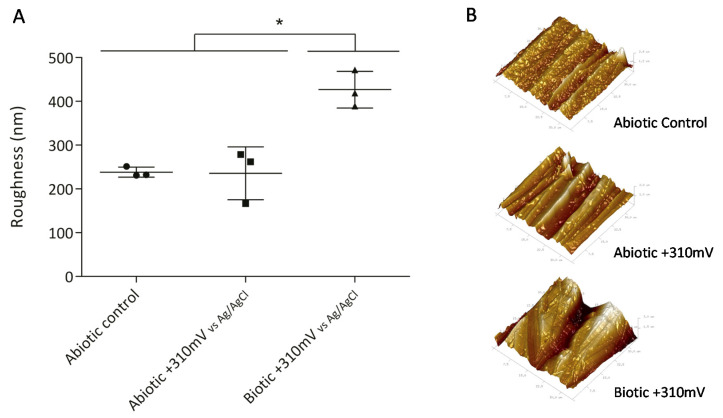
Roughness of SS electrodes (second experiment), measured by atomic force microscopy (AFM). Asterisks represent significant differences between treatments (*P* < 0.05). (**A**) Average roughness of SS electrodes with standard deviation and (**B**) 3D Atomic Force Microscope (AFM) images of SS electrodes obtained over an area of 35 µm × 35 µm.

To determine whether the significant weight loss observed under the +310 mV (vs. Ag/AgCl) potential was due to the effect of the electrochemically enriched bacteria or the potential itself, an abiotic control experiment was conducted. Figure 2B shows that the maximum weight loss, which occurred in the SS electrodes poised at +310 mV (vs. Ag/AgCl) under biotic conditions, was significantly different from the corrosion produced under abiotic conditions both with and without the overpotential (*P* < 0.05). This result suggests that the observed deterioration was associated more with the enrichment of specific bacteria than with abiotic corrosion. Besides, AFM analysis of the electrodes showed that the applied potential of +310 mV (vs Ag/AgCl) significantly increased the surface roughness, probably due to electrode corrosion (Figure 3).

Indeed, biocorrosion of SS has been linked with the action of autotrophic metabolism [36,37,38]. This type of corrosion is frequently associated with the ennoblement of the OCP involving iron-oxidizing bacteria, manganese-oxidizing bacteria [36,38], and, more recently, electro-autotrophic bacteria able to use an electrode (i.e., a SS coupon) as the electron donor to reduce oxygen [37,39,40]. Furthermore, the ennoblement of SS plates exposed to natural seawater typically shifts the OCP by +200 to +300 mV (vs. Ag/AgCl), bringing it closer to the pitting corrosion potential [37]. This microbially driven phenomenon results in the initiation of SS pitting and crevice corrosion. Pitting corrosion of 316L SS coupons exposed to seawater has been reported under an applied potential ranging from +500 to +600 mV (vs. Ag/AgCl) after a few days of immersion [41]. Therefore, the significant differences in weight loss (Figure 2) and roughness (Figure 3) exhibited by the coupons exposed to the +310 mV (vs. Ag/AgCl) potential, in comparison to those produced the other tested conditions, may be linked with the enrichment for particular bacterial groups that could catalyze electron transfer, rather than with the applied potential itself.

Under the tested conditions, no significative damage was observed on SS electrodes polarized at +100 mV (vs. Ag/AgCl) that correspond to OCP reported for 316L SS under marine conditions after 10 days [23]. These results suggest that SS is a good candidate to be used as electrode material for the scaling-up of METs in marine environments. Hence, this finding opens the opportunity to explore long-term evaluation of this material in marine METs involving biocathodic reactions.

### 3.2. Bacterial Enrichment Using Overpotentials

To corroborate the microbial community development on the tested electrodes, epifluorescence microscopy and SEM were performed (Supplementary Materials Figures S1 and S2). Microscopic analysis revealed the presence of microorganisms attached to the surface of the electrode, with the presence of extracellular polymeric-like substances (Figure S2). These results confirm early biofilm development on all the coupons.

During the experiment, three different enrichment levels were expected because of the experimental design, which would be influenced by (i) the effect of water storage (bottle effect), (ii) the effect of the SS surface (i.e., material composition and roughness), and (iii) the effect of the overpotential application. The storage conditions and the surface were transversal in all the conditions studied, allowing the isolation of the specific effect of microbial enrichment upon application of the overpotential.

**Figure 4:**
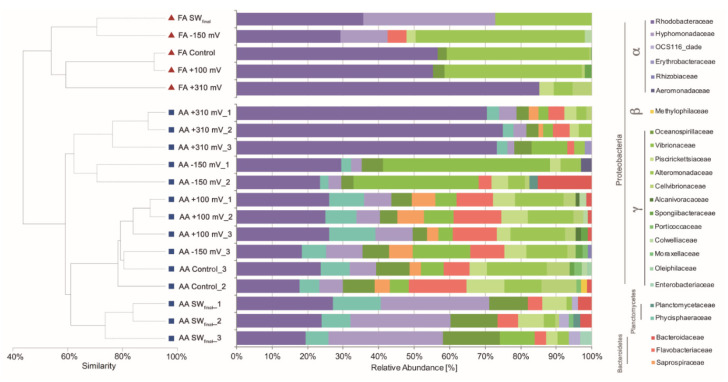
Cluster and relative abundance family-level taxonomic analysis of the bacterial communities associated with each treatment.

To evaluate the effect of the applied potentials on specific microbial enrichment, the bacterial community developed under each treatment was characterized at the end of the experiment using its 16S rRNA gene profiles. Cluster analysis based on the Bray–Curtis similarity coefficients obtained from both FA and AA datasets indicated a similar and consistent pattern, with the samples exposed at +310 mV (vs. Ag/AgCl) being the most different in each dataset (Figure 4), with 65% and 80% similarity, respectively. Differences were supported by SIMPROF (Pi 4,114, *P* < 0.05) and ANOSIM (R: 0.718, *P* < 0.05) analyses.

Cluster analyses were complemented with taxonomic assignation to the family level of the main OTUs detected by cloning (i.e., FA) and AA. In the case of the samples exposed at +310 mV (vs. Ag/AgCl), the Rhodobacteraceae bacterial family was highly enriched from a natural inoculum of seawater without any nutrients added. Both techniques, FA and AA, gave similar results in terms of relative abundance of Rhodobacteraceae in the +310 mV (vs. Ag/AgCl) samples, with an average relative abundance of 81.80% by FA and 69.31% (±2.81) by AA.

On the other hand, the +100 mV, −150 mV (vs. Ag/AgCl) polarization, and control treatments resulted in similar bacterial compositions (Figure 4), suggesting that the effect of applying +100 mV, −150 mV (vs. Ag/AgCl) to an electrode, on the enrichment of specific marine bacteria, was not greater than the effect generated by the material itself. *Sulfitobacter* and *Glaciecola*, two of the most abundant microbial genera identified in the +100 mV (vs. Ag/AgCl) and −150 mV (vs. Ag/AgCl) samples (Table 1), were previously identified as the most abundant genera in microbial communities developed over SS surfaces [42]. In the case of the control, wherein no potential was applied, typical microbial genera previously associated with electroactive microbial communities were identified. When exposed to seawater, the 316L SS surface potential naturally reached +100 mV (vs. Ag/AgCl) after 10 days [23], and as is shown in Figure 4, the control and +100 mV (vs. Ag/AgCl) treatments resulted in similar microbial community compositions. These results suggest that microbial settling in natural complex environments, as well as in reactors run under controlled laboratory conditions [18,19,20,21], depends on the value of the SS electrode potential.

### 3.3. Metabolic Inference of Electrochemically Enriched Bacterial Communities

To gain more information about the microbial community identified in each sample, the most abundant genera were characterized, identifying their metabolisms and the environments in which they have been found, according to previous reports in the literature. Table 2 lists, in decreasing order of abundance, the most abundant genera of each family that were identified for each treatment. Furthermore, the similarity between the sequences obtained by FA and AA, associated with each gender, was analyzed. All pairs of sequences had similarities of >80% and were related to the same bacterial genera, confirming the correspondence between a fingerprinting technique (such as FA) and AA analysis [43,44].

For the discussion of the relative abundance of the most abundant microbial genera identified in each condition, only the AA analyzes were used. The reason for this is due to the low number of clones obtained by condition, which generates an oversize of the relative abundance values of the genera identified by the clones in each condition (Table S2). The genera with higher relative abundances were *Roseobacter*, *Pheobacter*, and *Sulfitobacter*, with relative abundances of 16.8%, 10.5%, and 9.9%, respectively (Table 1). These genera showed the highest relative abundances in the +310 mV (vs. Ag/AgCl) samples. All of these genera have been related to autotrophic microorganisms and reported in electrochemically active microbial communities [42,45,46,47]. Other genera that have not been previously implicated in biocorrosion of SS, including *Aestuariicella*, *Lewinella*, and *Spongiibacter*, were also detected, with relative abundances of 2.43%, 3.18%, and 0.5%, respectively (Table 1). The majority of the genera shown in Table 2 have been previously associated with electroactive microbial communities developed on SS plates or electrodes [42,45,48,49,50,51,52,53,54,55,56,57,58].

As described in Materials and Methods, the potential of −150 mV (vs. Ag/AgCl) was selected as a representative midpoint potential capable to enrich for reported marine EAB from marine sediments [9]. The groups of microorganisms reported by Rowe et al. (2015) at −150 mV (vs. Ag/AgCl) were not found in our experiments. Nevertheless, *Sulfitobacter* and *Glaciecola* have been reported as the predominant bacteria in electroactive microbial communities on SS electrodes [42] (Table 2), and these were identified as two of the most abundant microbial genera enriched on the electrodes polarized at −150 mV (vs. Ag/AgCl) in our experiments (Table 1).

Poised potentials around +100 mV (vs. Ag/AgCl) have been identified as selective pressures for specific microbial communities mostly composed by *Marinobacter*, *Chromatiaceae*, and *Labrenzia* (MCL). This particular community, identified as a MCL-biocathode, has been reported to be capable of using the electrons supplied by the electrochemical system to drive CO_2_ fixation and O_2_ reduction [20,59,60]. Although *Marinobacter* and *Labrenzia* were not the most abundant genera identified in our electrodes tested over +100 mV (vs. Ag/AgCl), the abundances of both genera were significantly higher in comparison with their abundances in the other samples (Figure S3). It is important to highlight that the aforementioned MCL-biocathode cluster was obtained using marine sediment as the inoculum [61]. In this study, electrochemical enrichment was accomplished directly from natural seawater. In consequence, differences in bacterial composition and abundance between these experiments were expected.

*Roseobacter* and *Sulfitobacter* were two of the most abundant genera identified in the +310 mV (vs. Ag/AgCl) samples (Table 1). Both groups of microorganisms have been identified as EAB with high efficiency in the catalysis of the oxygen reduction reaction [46]. As indicated in Materials and Methods, this overpotential has been related to the oxygen reduction reaction [24,62]. From a bioenergetic perspective, with higher potentials closer to the theoretical reduction potential of oxygen (i.e., +600 mV vs. Ag/AgCl at pH = 7 and pO_2_ = 0.2 bar), aerobic microorganisms gain less energy [24]. Using an inoculum collected from a wastewater treatment plant, Ter Heijne et al. (2010) studied the effect of positive overpotentials (+50 mV, +150 mV, and +250 mV vs. Ag/AgCl) on the development of oxygen-reducing biocathodes. While the biocathodes polarized at +50 mV and +150 mV (vs. Ag/AgCl) produced current during the first day after inoculation, that polarized at +250 mV (vs. Ag/AgCl) produced no current until day 15, probably due to slow bacterial growth and a longer start-up time [24]. On the other hand, in a bioelectrochemical study of *Candidatus* Tenderia electrophaga (belonging to the Chromatiaceae family), Eddie et al. (2017) showed that by applying a potential as positive as +470 mV (vs. SHE) or +250 mV (vs. Ag/AgCl), there was an increase in the expression of genes related with nitrate and oxygen reduction [40]. Luo and Moran (2014) [63] reported that about half of the marine *Roseobacter* clade present genes related to the nitrate reduction process, and as indicated above, *Sulfitobacter* and *Roseobacter* can reduce oxygen using electrons from a polarized electrode [46]. Our results showed that by applying a positive potential as high as +310 mV (vs. Ag/AgCl) to SS electrodes exposed to natural seawater, it was possible to establish a high enrichment pressure for enriching autotrophic oxygen-reducing EAB such as *Sulfitobacter* and *Roseobacter*.

The presence of autotrophic oxygen-reducing EAB has been related to the acceleration of biocorrosion processes [37,39,40]. The highest abundances of *Sulfitobacter* and *Roseobacter* were found in the +310 mV (vs. Ag/AgCl) treatment, and at the same time, this condition resulted in significant differences in weight loss (Figure 2) and roughness (Figure 3) in comparison with those produced under the other tested conditions. Therefore, the results of this research suggest that high abundances of *Sulfitobacter* and *Roseobacter* are directly related to electrode deterioration, specifically the biocorrosion of 316L SS.

## 4. Conclusions

Electrochemical bacterial enrichment from natural seawater without nutrients amended was successfully achieved for the first time to the best of our knowledge. Under the tested conditions, no significant damage was observed on SS electrodes poised at up to +100 mV (vs. Ag/AgCl). This finding opens the opportunity for long-term evaluation of SS as electrode material in marine METs. On the other hand, a significant selection for putative EAB within the *Roseobacter* and *Sulfitobacter* genera present in a marine inoculum was observed upon applying an overpotential of +310 mV (vs. Ag/AgCl), and the resultant microbial community accelerated corrosion, compromising the lifetimes of the SS electrodes. Finally, further attention should be given to the aforementioned bacterial groups as potential biological drivers of pitting corrosion in 316L SS in marine environments.

## Figures and Tables

**Table 1:** Relative abundance (%) of most-abundant genre identified by amplicon analysis (AA) in each sample (+100 mV, −150 mV, +310 mV vs. Ag/AgCl and Control).

Family	Genus	Relative Abundance (%)			
		AA			
		+100 mV	−150 mV	+310 mV	Control
Rhodobacteraceae	Roseobacter	4.3	3.9	34.0	6.4
	Phaeobacter	6.0	4.8	18.8	5.9
	Sulfitobacter	7.2	7.9	15.2	3.5
	Ruegeria	1.3	1.0	0.5	1.2
	Labrenzia	1.1	0.0	0.1	0.1
Vibrionaceae	Vibrio	4.0	3.3	3.8	5.0
	Photobacterium	0.1	0.0	0.0	0.0
	Aliivibrio	0.0	0.0	0.0	0.0
Hyphomonadaceae	Hyphomonas	4.3	3.4	3.6	5.1
	Maricaulis	1.0	0.3	0.7	0.8
Flavobacteriaceae	Muricauda	5.3	2.1	1.7	3.7
	Maribacter	0.0	0.0	0.0	0.0
	Cellulophaga	0.0	0.0	0.0	0.0
Alteromonadaceae	Alteromonas	0.6	0.1	2.5	11.5
	Glaciecola	7.3	5.9	0.0	0.6
	Marinobacter	2.9	0.2	0.0	0.2
Phycisphaeraceae	Plantomycete	10.3	4.2	2.2	6.6
Oceanospirillaceae	Neptuniibacter	3.7	4.3	3.3	6.5
	Amphritea	0.1	0.0	0.0	0.0
	Oleibacter	0.7	0.1	0.0	1.1
Piscirickettsiaceae	Methylophaga	5.1	3.3	2.1	6.9
Cellvibrionaceae	Aestuariicella	2.3	0.8	0.5	4.5
Saprospiraceae	Lewinella	5.5	1.7	1.3	1.7
Bacteroidaceae	Bacteroides	1.1	4.6	0.0	0.5
Colwelliaceae	Colwellia	0.9	0.0	0.0	0.7
Spongiibacteraceae	Spongiibacter	0.5	0.5	0.0	0.6

**Table 2:** Similarity analysis between sequences obtained by fragment analysis (FA) and amplicon analysis (AA), and their metabolisms and the environments wherein have been reported. The similarity between sequences obtained by AA and FA is expressed as % similarity.

Family	Most Abundant Genus Identified by AA	% Similarity	Most Abundant Genus Identified by FA	Metabolism and Environments Wherein They Have Been Reported
Rhodobacteraceae	*Roseobacter*	90	*Roseobacter*	1. Aerobic anoxygenic photosynthesis [47].
				2. Identified as primary colonizers on surfaces exposed to seawater [64].
				3. Reported as EAB with high efficiency in the catalysis of the oxygen reduction reaction [46].
	*Pheobacter*	93	*Pheobacter*	1. Reported in polarized stainless steel cathode [45].
				2. Association with *Roseobacter* during primary colonization [45].
				3. Aerobic anoxygenic photosynthesis [45].
	*Sulfitobacter*	80	*Sulfitobacter*	1. Reported on the bacterial communities associated with the early stages of marine corrosion of carbon steel [65].
				2. Reported as EAB with high efficiency in the catalysis of the oxygen reduction reaction [46].
				3. Has been found in both anodic and cathodic biofilms [42].
Vibrionaceae	*Vibrio*	-	-	1. Reported in graphite bioanodes present in bioelectrochemical systems [54].
Hyphomonadaceae	*Hyphomonas*	99	*Hyphomonas*	1. Identified in graphite biocathodes present in an MFC [66].
	*Maricaulis*	90	*Maricaulis*	1. Identified as a typical bacterioplankton in marine ecosystems [67].
				2. Reported as the primary colonizer in biofilm developed on stainless steel [68].
Flavobacteriaceae	*Muricauda*	87	*Muricauda*	1. Reported in a biocathode microbial community [52].
Alteromonadaceae	*Alteromonas*	85	*Alteromonas*	1. Reported on electrochemically active biofilms [51].
				2. Chemo-heterotrophic halophytes [51].
	*Glaciecola*	87	*Glaciecola*	1. Aerobic chemo-heterotrophic bacteria [69].
				2. Reported as predominant bacteria in electroactive biofilms on stainless steel electrodes [42].
Phycisphaeraceae	Planctomycetes	-	-	1. Reported in marine phototrophic consortia that can transfer electrons to electrodes in response to reductive stress [70].
Oceanospirillaceae	*Neptuniibacter*	-	-	1. Reported in microbial community associated with stainless steel coupons [56].
Piscirickettsiaceae	Methylophaga	-	-	1. Reported in stainless steel and carbon steel cathodes [57].
				2. Has been reported to reduce nitrate to nitrite [71].
				3. Halophilic methylotrophic metabolism [72].
Cellvibrionaceae	Aestuariicella	-	-	1. Aliphatic hydrocarbon-degrading bacterium [73].
				2. Not yet reported in biofilms associated with stainless steel [73].
Saprospiraceae	Lewinella	-	-	1. Isolated from marine sediment [74].
				2. Not yet reported in biofilms associated with stainless steel [74].
Bacteroidaceae	Bacteroides	-	-	1. Reported on stainless steel electrodes [58].
Colwelliaceae	Colwellia	89	Colwellia	1. Reported on carbon steel cathodes [71].
				2. Identified in a marine biofilm exposed to high concentration of nitrate [71].
Spongiibacteraceae	Spongiibacter	92	Spongiibacter	1. Halophilic marine bacterium [75].
				2. Not yet reported in biofilms associated with stainless steel [75].

## Supplementary Materials

The following are available online at https://www.mdpi.com/1996-1944/13/10/2327/s1, Figure S1: Epifluorescence microscopy of each treatment, using acridine orange 0.1% w/v. When an acridine orange binds with DNA, it will exhibit a green color, and when it binds with RNA, it will exhibit a red color. (A) +310 mV vs. Ag/AgCl, (B) −150 mV vs. Ag/AgCl, (C) +100 mV vs. Ag/AgCl, and (D) Control. All images are in 10× magnification, Figure S2: Scanning Electron Microscopy of each treatment. (A) +310 mV vs. Ag/AgCl, (B) −150 mV vs. Ag/AgCl, (C) +100 mV vs. Ag/AgCl, and (D) Control. Bar scale represents a size of 2 µm. The magnifications of these images are 13,900×, Figure S3: Relative abundance (%) of *Marinobacter sp*. and *Labrenzia sp*. in each sample, Table S1: Accession number of the clone sequences reported in this investigation and the most closely related organisms, with its accession number, Table S2: Relative abundance (%) of most abundant genre identified by amplicon analysis (AA) and fragment analysis (FA) in each sample (+100mV, −150mV, +310mV vs. Ag/AgCl and Control).
