# Supplementary material for: Electrochemical Bacterial Enrichment from Natural Seawater and Its Implications in Biocorrosion of Stainless-Steel Electrodes
Source: Materials (Basel). 2020 May 19;13(10):2327. doi: 10.3390/ma13102327 (PMC7288148; doi:10.3390/ma13102327)
Supplement: Supplementary file 1 [file materials-13-02327-s001.pdf]

materials

Supplementary Materials

Electrochemical Bacterial Enrichment from Natural Seawater and Its Implications in Biocorrosion of Stainless-Steel Electrodes

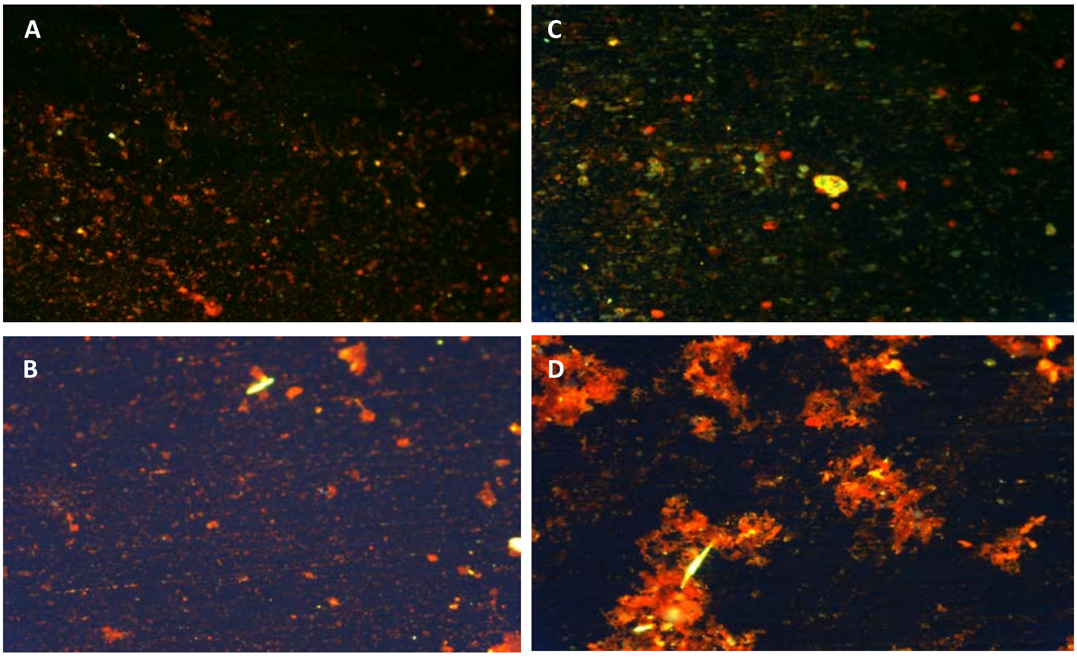

Figure S1. Epifluorescence microscopy of each treatment, using acridine orange 0.1% w/v. When an acridine orange binds with DNA, it will exhibit a green color, and when it binds with RNA, it will exhibit a red color. (**A**): +310 mV vs Ag/AgCl, (**B**): −150 mV vs Ag/AgCl, (**C**): +100 mV vs Ag/AgCl, and (**D**): Control. All images are in 10× magnification.

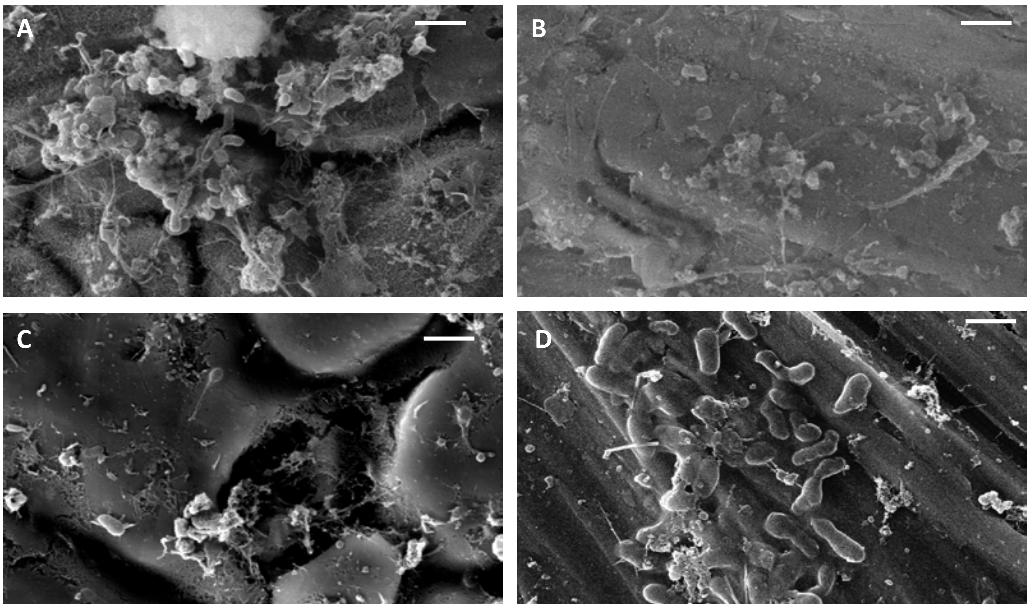

Figure S2. Scanning Electron Microscopy of each treatment. (**A**): +310 mV vs Ag/AgCl, (**B**): −150 mV vs Ag/AgCl, (**C**): +100 mV vs Ag/AgCl, and (**D**): Control. Bar scale represents a size of 2 μm. The magnifications of these images are 13,900×.

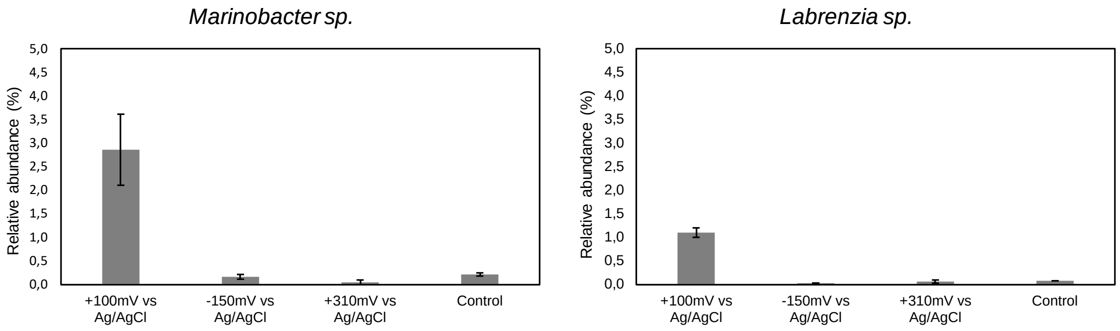

Figure S3. Relative abundance (%) of *Marinobacter* sp. and *Labrenzia* sp. in each sample.

Table S1. Accession number of the clone sequences reported in this investigation and the most closely related organisms, with its accession number.

Condition	Accession N° (for This Research)	Most Closely Related Organisms	Accession N°
+310 mv (vs Ag/AgCl)	MT026239	*Phaeobacter* sp.	MN099587.1
+310 mv (vs Ag/AgCl)	MT026240	*Methylophaga* sp.	KC295387.1
+310 mv (vs Ag/AgCl)	MT026241	*Roseobacter* sp.	AF098493.1
+310 mv (vs Ag/AgCl)	MT026242	*Alteromonas* sp.	JX022750.1
+310 mv (vs Ag/AgCl)	MT026243	*Halioxenophilus aromaticivorans*	AB809162.1
+310 mv (vs Ag/AgCl)	MT026244	*Pseudophaeobacter* sp.	MK737663.1
+310 mv (vs Ag/AgCl)	MT026245	*Phaeobacter caeruleus*	HM031996.1
+310 mv (vs Ag/AgCl)	MT026246	*Leisingera* sp.	CP038234.1
+310 mv (vs Ag/AgCl)	MT026247	Uncultured bacterium clone methane	GU584300.1
+310 mv (vs Ag/AgCl)	MT026248	*Roseobacter* sp.	JQ661253.1
+310 mv (vs Ag/AgCl)	MT026249	*Phaeobacter* sp.	HE818248.1
−150 mv (vs Ag/AgCl)	MT026250	Uncultured Piscirickettsiaceae bacterium	DQ234105.2
−150 mv (vs Ag/AgCl)	MT026251	*Roseobacter* sp.	AY576690.1
−150 mv (vs Ag/AgCl)	MT026252	*Ruegeria* sp.	MN099589.1
−150 mv (vs Ag/AgCl)	MT026253	Uncultured *Colwellia* sp.	JN860307.1
−150 mv (vs Ag/AgCl)	MT026254	*Hyphomonas* sp.	CP017718.1
−150 mv (vs Ag/AgCl)	MT026255	*Marinobacterium stanieri*	NR_024699.1
Control	MT026256	*Alteromonas stellipolaris*	LR218097.1
Control	MT026257	*Glaciecola* sp.	JX310209.1
Control	MT026258	Uncultured bacterium clone	KX177808.1
Control	MT026259	Spongiibacter *marinus*	NR_118015.1
Control	MT026260	Uncultured *Alteromonas* sp.	KC917978.1
Control	MT026261	*Phaeobacter* sp.	FJ436728.1
Control	MT026262	*Phaeobacter gallaeciensis*	CP015124.1
Control	MT026263	*Aestuariicella hydrocarbonica*	NR_135890.1
Control	MT026264	*Phaeobacter* sp.	FJ436729.1
Control	MT026265	*Pseudoalteromonas* sp.	AM162590.1
Counter	MT026266	*Alteromonas* sp.	JX022750.1
Counter	MT026267	*Alteromonas macleodii*	CP018321.1
Counter	MT026268	*Vibrio* sp.	LC506146.1
Counter	MT026269	*Hyphomonas* sp.	KC295391.1
Counter	MT026270	*Phaeobacter* sp.	FJ436728.1
Counter	MT026271	*Roseobacter* sp.	AY576690.1
Counter	MT026272	*Phaeobacter* sp.	FJ014980.1
Counter	MT026273	*Sulfitobacter* sp.	EU864265.1
Initial Sea water	MT026274	Uncultured Flavobacteria	AM279180.1

Initial Sea water	MT026275	*Planktomarina temperata*	NR_125550.1
Initial Sea water	MT026276	Uncultured Flavobacteria	AM279180.1
Initial Sea water	MT026277	*Winogradskyella* sp.	CP019332.1
Initial Sea water	MT026278	Uncultured Flavobacteria	EF202334.1
Initial Sea water	MT026279	*Loktanella* sp.	MK737661.1
Initial Sea water	MT026280	Uncultured bacterium clone	MK176136.1
Initial Sea water	MT026281	*Planktomarina temperata*	NR_125550.1
Initial Sea water	MT026282	Uncultured Pseudomonas sp.	KP453918.1
Initial Sea water	MT026283	Uncultured bacterium clone	JQ198943.1
Final Sea water	MT026284	*Sulfitobacter* sp.	MF600215.1
Final Sea water	MT026285	*Hyphomonas* sp.	KC295391.1
Final Sea water	MT026286	Uncultured bacterium clone	JQ200192.1
Final Sea water	MT026287	*Maricaulis maris*	CP000449.1
Final Sea water	MT026288	Uncultured *Hyphomonas* sp.	FJ425626.1
Final Sea water	MT026289	*Pseudoalteromonas* sp.	KF009870.1
Final Sea water	MT026290	*Maricaulis maris*	CP000449.1
Final Sea water	MT026291	*Roseobacter* sp.	AJ534238.1
Final Sea water	MT026292	*Hyphomonas* sp.	KC295391.1

Table S2. Relative abundance (%) of most abundant genre identified by Amplicon Analysis (AA) and Fragment Analysis (FA) in each sample (+100 mV, −150 mV, +310 mV vs Ag/AgCl and Control).

Family	Genus	Relative Abundance (%)							
		AA				FA			
		+100 mV	−150 mV	+310 mV	Control	+100 mV	−150 mV	+310 mV	Control
Rhodobacteraceae	Roseobacter	4.3	3.9	34.0	6.4	0.0	18.2	37.0	0.0
	Phaeobacter	6.0	4.8	18.8	5.9	55.2	0.0	44.8	56.0
	Sulfitobacter	7.2	7.9	15.2	3.5	0.0	0.0	0.0	0.0
	Ruegeria	1.3	1.0	0.5	1.2	0.0	4.3	0.0	0.0
	Labrenzia	1.1	0.0	0.1	0.1	0.0	0.0	0.0	0.0
Vibrionaceae	Vibrio	4.0	30.3	3.8	5.0	0.0	0.0	0.0	0.0
	Photobacterium	0.1	0.0	0.0	0.0	0.0	0.0	0.0	0.0
	Aliivibrio	0.0	0.0	0.0	0.0	0.0	0.0	0.0	0.0
Hyphomonadaceae	Hyphomonas	4.3	3.4	3.6	5.1	0.0	13.3	0.0	0.0
	Maricaulis	1.0	0.3	0.7	0.8	0.0	0.0	0.0	0.0
Flavobacteriaceae	Muricauda	5.3	2.1	1.7	3.7	0.0	0.0	0.0	0.0
	Maribacter	0.0	0.0	0.0	0.0	0.0	5.3	0.0	0.0
	Cellulophaga	0.0	0.0	0.0	0.0	0.0	0.0	0.0	0.0
Alteromonadaceae	Alteromonas	0.6	0.1	2.5	11.5	32.0	47.6	5.2	37.0
	Glaciecola	7.3	5.9	0.0	0.6	6.3	0.0	0.0	2.6
	Marinobacter	2.9	0.2	0.0	0.2	0.0	0.0	0.0	0.0
Phycisphaeraceae	Plantomycete	10.3	4.2	2.2	6.6	0.0	0.0	0.0	0.0
Oceanospirillaceae	Neptuniibacter	3.7	4.3	3.3	6.5	3.2	0.0	0.0	2.3
	Amphritea	0.1	0.0	0.0	0.0	0.0	0.0	0.0	0.0
	Oleibacter	0.7	0.1	0.0	1.1	0.0	0.0	0.0	0.4
Piscirickettsiaceae	Methylophaga	5.1	3.3	2.1	6.9	0.0	2.6	3.8	0.0
Cellvibrionaceae	Aestuariicella	2.3	0.8	0.5	4.5	0.7	0.0	5.2	0.3
Saprospiraceae	Lewinella	5.5	1.7	1.3	1.7	0.0	0.0	0.0	0.0
Bacteroidaceae	Bacteroides	1.1	4.6	0.0	0.5	0.0	0.0	0.0	0.0
Colwelliaceae	Colwellia	0.9	0.0	0.0	0.7	0.0	1.9	0.0	0.0
Spongiibacteraceae	Spongiibacter	0.5	0.5	0.0	0.6	2.1	0.0	0.0	0.4

© 2020 by the authors. Submitted for possible open access publication under the terms and conditions of the Creative Commons Attribution (CC BY) license (http://creativecommons.org/licenses/by/4.0/).
